# Creation of a Student-Run Medical Education Podcast: Tutorial

**DOI:** 10.2196/29157

**Published:** 2021-07-08

**Authors:** Kevin John Milligan, Robert Scott Daulton, Zachary Taylor St Clair, Madison Veronica Epperson, Rachel Mackenzie Holloway, Jeffrey David Schlaudecker

**Affiliations:** 1 University of Cincinnati College of Medicine Cincinnati, OH United States; 2 Department of Otolaryngology-Head and Neck Surgery University of Michigan Ann Arbor, MI United States; 3 Department of Family and Community Medicine University of Cincinnati College of Medicine Cincinnati, OH United States

**Keywords:** podcast, medical student, near-peer, medical education

## Abstract

**Background:**

Podcasting has become a popular medium for medical education content. Educators and trainees of all levels are turning to podcasts for high-quality, asynchronous content. Although numerous medical education podcasts have emerged in recent years, few student-run podcasts exist. Student-run podcasts are a novel approach to supporting medical students. Near-peer mentoring has been shown to promote medical students’ personal and professional identity formation. Student-run podcasts offer a new medium for delivering near-peer advice to medical students in an enduring and accessible manner.

**Objective:**

This paper describes the creation of the *UnsCripted Medicine Podcast*—a student-run medical education podcast produced at the University of Cincinnati College of Medicine.

**Methods:**

The planning and preparatory phases spanned 6 months. Defining a target audience and establishing a podcast mission were key first steps. Efforts were directed toward securing funding; obtaining necessary equipment; and navigating the technical considerations of recording, editing, and publishing a podcast. In order to ensure that high professionalism standards were met, key partnerships were created with faculty from the College of Medicine.

**Results:**

The *UnsCripted Medicine Podcast* published 53 episodes in its first 2 years. The number of episodes released per month ranges from 0 to 5, with a mean of 2.0 episodes. The podcast has a Twitter account with 217 followers. The number of listeners who subscribed to the podcast via Apple Podcasts grew to 86 in the first year and then to 218 in the second year. The show has an average rating of 4.8 (out of 5) on Apple Podcasts, which is based on 24 ratings. The podcast has hosted 70 unique guests, including medical students, resident physicians, attending physicians, nurses, physicians’ family members, graduate medical education leadership, and educators.

**Conclusions:**

Medical student–run podcasts are a novel approach to supporting medical students and fostering professional identity formation. Podcasts are widely available and convenient for listeners. Additionally, podcast creators can publish content with lower barriers of entry compared to those of other forms of published content. Medical schools should consider supporting student podcast initiatives to allow for near-peer mentoring, augment the community, facilitate professional identity formation, and prepare the rising physician workforce for the technological frontier of medical education and practice.

## Introduction

Podcasting has become an established and increasingly popular means of delivering asynchronous educational content and entertainment. The potential for podcasts to be a platform for educational content was identified in the early 2000s [1,2]. Over the past decade, several medical education podcasts have risen to prominence and have been formally and informally included in both undergraduate and graduate medical education. For example, nearly 90% of emergency medicine residents have reported listening podcasts at least once per month to stay up to date with relevant literature [3]. Additionally, a small pilot study showed that podcasting was as effective as conventional lectures in increasing anesthesia residents’ knowledge of electroencephalogram interpretation [4]. Medical students have also been identified as significant consumers of medical education podcasts [5]. Listeners have reported that podcasts deliver quality content, allow for personalized learning, and are more convenient than traditional print media [5]. Furthermore, podcasting platforms offer several benefits to content creators and educators.

A key benefit of podcast hosting is a lower barrier of entry compared to those of other forms of digital media. Video media production often requires expensive and specialized equipment; however, podcast creators can quickly and affordably produce quality content for their listeners. With regard to assisting physicians and medical educators in creating their own podcasts, several articles have been published describing the process of starting a medical education podcast [1,6-9]. However, all of the published literature regarding medical education podcast creation has been written by resident physicians, attending physicians, or professional medical educators; medical students’ voices are absent.

Student-run podcasts are a novel approach to supporting medical students. Student-run podcasts can augment the sense of community within a medical school and introduce new opportunities for near-peer mentoring. For the purposes of this paper, near-peer mentoring is defined as a mentoring relationship in which a more senior learner (≥1 year higher) provides guidance and support to a new junior learner to enable the new student to navigate their own education [10]. Near-peer mentoring has been shown to promote medical students’ personal and professional identity formation [10]. Student-run podcasts offer a new medium for delivering near-peer mentoring to medical students in an enduring and accessible way. Very few medical student–run podcasts exist, despite their potential to facilitate community engagement and empower learning. This paper describes the successful creation of the *UnsCripted Medicine Podcast*—a medical student–run podcast produced at the University of Cincinnati College of Medicine (COM).

## Methods

### Overview of the Podcast

Our podcast project was formed to address a perceived need for increasing the amount of near-peer mentoring in the COM community. Through discussions with medical students across all 4 years of training, the creators of the *UnsCripted Medicine Podcast* found that medical students frequently seek informal advice from more experienced senior peers. Additionally, many students attended formal events involving expert panels that were comprised of senior medical students to prepare for upcoming courses and clerkships. Students reported an affinity for near-peer advice and mentoring resulting from the collegiality of the relationships they formed and a general trust of senior medical students resulting from their recent direct exposure to relevant experiences. Despite their popularity and perceived benefits, formal near-peer mentoring events occur at infrequent intervals, and informal relationships rely on both the initiative of the mentee and the availability of a sufficient number of willing mentors.

Podcasting was identified as an alternative and more accessible medium for providing near-peer mentoring and support to a larger and more diverse cohort of students. As such, 4 students who were interested in addressing this identified need engaged in conversations about the steps required to launch a podcast hosted by and for medical students. The early planning and discussions between these future hosts focused primarily on the target audience, mission and content, equipment and costs, technical skills, and professionalism.

### Launch Timeline

**Figure 1:**
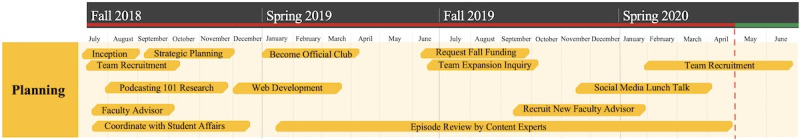
A timeline of key milestones in the creation of the *UnsCripted Medicine Podcast*.

Preliminary discussions, which started in July 2018, were followed by a 6-month preparatory phase. During this time, the podcast team engaged in strategic planning, performed a literature review, underwent technical training on audio recording and editing, developed a website with podcasting capabilities, and worked with COM leadership to ensure that administrative support and mentorship were available. The first episode was recorded in December 2018 and published in January 2019 (Figure 1).

### Target Audience

Identifying a target audience is the most crucial step in podcast creation [8]. It was intentionally determined that the podcast’s content would target medical students within the local COM community. To best provide relevant and valuable content for our audience, we emphasized the delivery of local content—all episodes involved a combination of students, residents, physicians, and educators from within the COM community. Although this approach may have had the effect of limiting the breadth of listenership, it provided a way to build connections and enrich the depth of the community within the academic medical center.

### Mission and Content

Podcasting is an open format that can accept any number of different views, perspectives, voices, and approaches to content creation. Although this is a great asset, open platforms pose unique challenges. Content creation must balance utility with entertainment and balance generalizability with specificity. Defining our mission—promoting student success through near-peer advice; uplifting the COM community by creating a broader sense of solidarity; and highlighting local clinicians, leaders, and educators—facilitated the creation of relevant and high-value content for our audience. At the core of our efforts was an emphasis on broadcasting diverse perspectives and creating an inclusive environment in which guests and hosts alike could share their unique stories. Through this mission, we sought to create a platform in which students could discuss their personal barriers and facilitators to academic and clinical success as well as relevant topics in wellness, humanism, and other domains that are not frequently discussed in the core curriculum.

The first 3 episodes were part of a series covering the US Medical Licensing Examination (USMLE) Step 1 exam. These episodes sought to provide general advice on how to approach the exam, included discussions of study schedules, and covered test day logistics. Given the efficacy of near-peer USMLE review courses, this was a natural starting point [11]. Due to its heightened relevance for preclinical medical students, this series had tremendous potential to quickly establish a large listenership. Subsequent episodes focused on academic success in preclinical coursework and subsequent core clinical clerkships. In these episodes, third-year and fourth-year medical students shared their experiences and success strategies. The recruitment of student guests focused equally on those who exhibited strong conversation skills and enthusiasm and those who demonstrated academic achievement. This was done to fulfill the stated goals of demystifying upcoming coursework and presenting multiple strategies for success through fluid and entertaining discussion. Episodes were recorded and released in accordance with the academic schedule, thereby providing just-in-time, near-peer advice for upcoming coursework. Upon the conclusion of the curricular series, episode content was expanded to other topics and themes (Textbox 1).

**Topics and themes of the *UnsCripted Medicine Podcast*.**

**Topics and themes**

- US Medical Licensing Examination preparation
- Curricular success
- Web-based shadowing
- *Digital Second Look* series of episodes
- Careers in academic medicine
- Humanism
- Personal finance
- Social media
- Premedicine and admissions
- Fireside chats (conversations with the College of Medicine faculty and leadership)

### Equipment and Costs

PCs were used for the recording and editing of all episodes. During initial in-person recording, we used a standard USB microphone. The COVID-19 pandemic necessitated a shift to remote recording, which required the purchase of additional USB microphones. The total costs associated with starting and operating the *UnsCripted Medicine Podcast* are detailed in Table 1.

The cost of entry could be a barrier for new podcast creators. Website hosting services and domain names are notable and recurring budget items. During the COVID-19 pandemic, remote recording software may add an additional expense. As medical students, our team has received partial funding from the COM Medical Student Association, which provides funding for student groups. Additional expenses were paid out of pocket by team members.

**Table 1:** Start-up costs for the launch of the *UnsCripted Medicine Podcast*.

Start-up requirements	Items and services used (cost; manufacturer)	Alternatives considered (cost; manufacturer)
Website and podcast hosting	Squarespace (US $144/year; Squarespace Inc)	Buzzsprout (US $144/year; Higher Pixels)
USB microphone^a^	Blue Microphones Yeti (US $129.99; Baltic Latvian Universal Electronics LLC)	Blue Microphones Snowball (US $69.99; Baltic Latvian Universal Electronics LLC)
Headphones^b^	Wired and wireless options (variable costs)	—^c^
Domain name	Squarespace (US $20/year; Squarespace Inc)	GoDaddy (variable; GoDaddy Inc)
Recording and editing software	GarageBand (free; Apple Inc)	Audacity (free)
Intro and outro music	YouTube Audio Library (free; Google LLC)	—
Logo	Freelance graphic designer (free)	—

^a^As a budget option, wired headphones with a microphone offer acceptable audio quality and are widely available. We discouraged the use of Bluetooth headphone microphones due to subjectively inferior audio quality.

^b^Headphone use during remote podcast recording is highly recommended to remove echoes.

^c^Not available. No alternatives were considered for this requirement.

### Technical Skills

Although podcasting offers a relatively low barrier of entry, it is important to note the technical skills required. Each step of the process—audio recording, editing, publishing, operating a website, and social media promotion—requires a unique skill set. Depending on their technological fluency, podcasting could present a steep learning curve for new podcast hosts. Numerous web-based resources exist to facilitate this learning [12-15].

At the beginning of podcast production, each team member was assigned to only 1 portion of the production process. One individual edited all of the episodes, another managed the website and publishing process, and a third distributed the episodes to the student body. This resulted in a streamlined production process and allowed team members to become proficient in their assigned task. Once a steady workflow was established, expertise was shared within the group to ensure that each team member could record, edit, and publish episodes independently. The ability to independently produce an episode from start to finish was an important step for ensuring the steady production of episodes regardless of any one student’s schedule or workload. This system also promoted sustainability because graduating students did not leave the podcast team without the necessary skills for carrying the project forward.

### Professionalism

Medical students are held to high standards of professionalism while in and out of the school, hospital, and clinic. For medical students who are interested in starting a podcast, there are important professionalism considerations. It is crucial to acknowledge that medical students are not yet physicians; they should therefore not represent themselves as such and must refrain from offering medical advice. Content published via podcasts creates a lasting digital footprint; accordingly, we avoided using offensive, derogatory, and explicit language.

Paramount to ensuring professionalism was our partnership with the COM administration. The Office of Student Affairs was closely involved with episode review in the first year of production. The COM faculty approved each episode prior to its public release and infrequently requested the omission or modification of content to ensure accuracy and freedom from professionalism concerns. After producing numerous episodes with close oversight, both the podcast team and COM administration agreed to transition to a self-regulatory system, which involved internal reviews as well as faculty consultation when appropriate.

## Results

**Figure 2:**
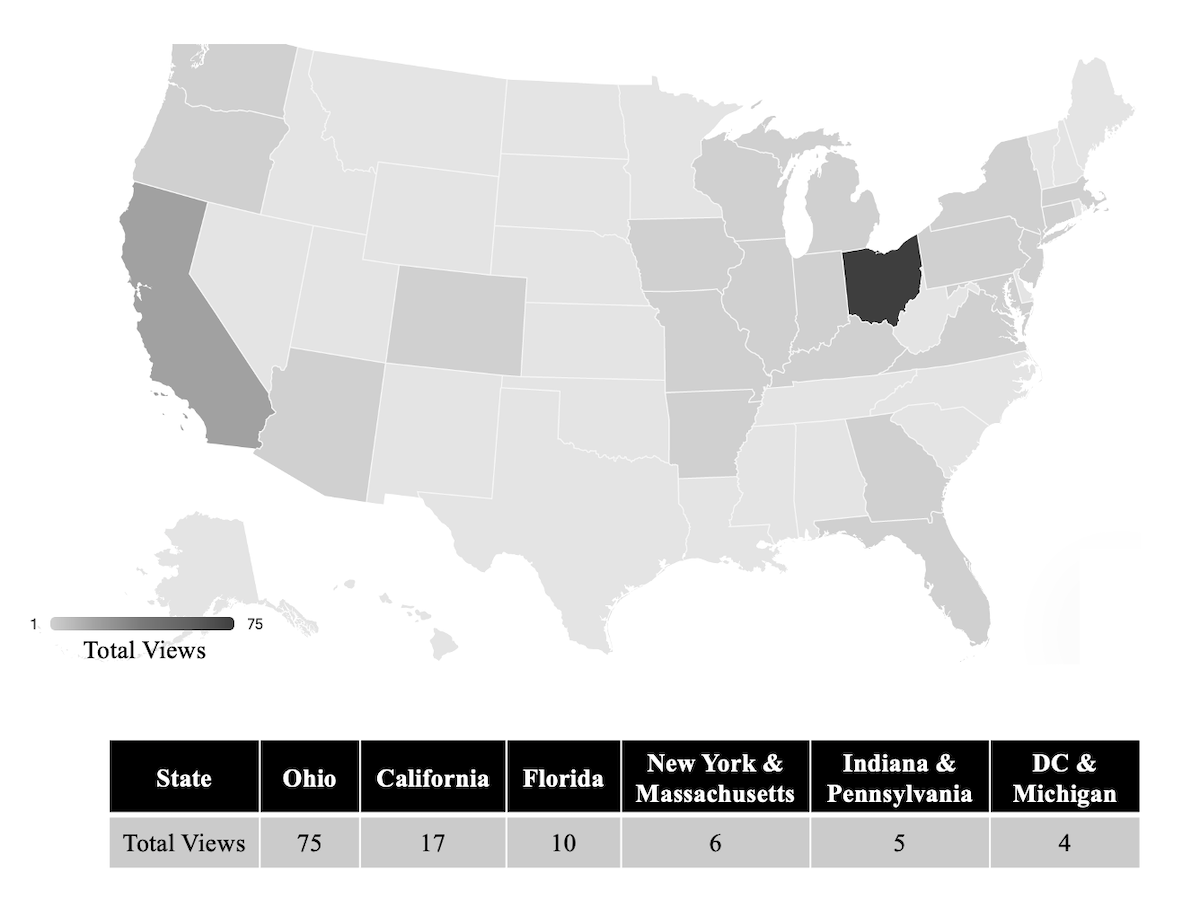
Geographic distribution of listeners of the *Digital Second Look* series of episodes. DC: District of Columbia.

The *UnsCripted Medicine Podcast* published 53 episodes in its first 2 years. The number of episodes released per month ranges from 0 to 5, with a mean of 2.0 episodes. The podcast has a Twitter account with 217 followers. The number of listeners who subscribed to the podcast via Apple Podcasts grew to 86 in the first year and then to 218 in the second year. The show has an average rating of 4.8 (out of 5) on Apple Podcasts, which is based on 24 ratings. The podcast has hosted 70 unique guests, including medical students, resident physicians, attending physicians, nurses, physicians’ family members, graduate medical education leadership, and educators. To characterize podcast listenership, the podcast’s geographical reach was analyzed over a 1-week period for a specific 4-episode series (*Digital Second Look*). Ohio had the most listeners (75/154, 48.7%), followed by California (17/154, 11%), Florida (10/154, 6.5%), New York (6/154, 3.9%), Massachusetts (6/154, 3.9%), and others (combined: 40/154, 26%; Figure 2).

Episode duration ranges from 6 minutes to 85 minutes. The mean episode duration is 46 minutes (SD 17 minutes). This is in line with our target length of <50 minutes as well as with the mean episode length for podcasts across all categories (41 minutes and 31 seconds) [16]. Overall, podcasts in the Medicine category have a median duration of about 26 minutes [16].

## Discussion

The *UnsCripted Medicine Podcast* was created to address the need for increasing the amount of near-peer mentoring. Through a close relationship with the COM faculty, students were able to launch a podcast with high standards of professionalism that provided near-peer advice on curricular blocks and rotations. Since its creation, the podcast has also served as a catalyst for fostering community engagement with a diverse array of guest hosts, including community pediatricians, assistant program directors, COM deans, COM faculty, professors from outside institutions, and students from each class. Pockets of the academic medicine campus that were previously disconnected have been connected through storytelling and mentorship. Further, as episode content begins to expand beyond local, COM-specific topics, the podcast team recognizes their growing potential to reach an increasingly comprehensive audience via discussions that apply more broadly to premedical and medical students across the country. The early analysis of a limited sample of episodes revealed that while listenership remains primarily local, the podcast has also reached small audiences from other parts of the country. Given the overall local nature of this podcast, the number and distribution of downloads may not be optimal metrics for gauging success. Instead, alternative metrics that assess community building and student well-being should be considered in future research.

Many structural elements were key to the podcast’s success. Building a team that spanned the spectrum of preclinical and clinical years was paramount to remaining in touch with the needs of the student body. The perspectives of preclinical medical students also enabled the podcast to successfully penetrate the premedical market through the *Admissions and Second Look* series. Furthermore, a team comprised of second-, third-, and fourth-year medical students established a framework to ensure podcast longevity; as 1 or more podcast hosts transition to residency, younger team members step into leadership roles to mentor the next generation of podcast leaders. Medical student–run podcasts provide a tremendous opportunity for professional identity formation. As such, the podcast team established a recruiting infrastructure that will enable medical students from each year to interface with the podcast as either hosts or guests. Social connections that were established through faculty mentors and other student organizations broadened the candidate pool for guests beyond immediate social circles. Placing an emphasis on local topics with high relevancy helped the podcast team establish a listenership within the target audience. The use of a hosting website that provided a Really Simple Syndication feed to major podcasting services ensured that listeners could conveniently listen to episodes by using their preferred podcast app. Podcast promotion via social media (ie, Twitter) helped the team rapidly disseminate new content and increased the podcast’s visibility to listeners outside of the COM community.

The *UnsCripted Medicine Podcast* faced a gamut of challenges while the team navigated podcasting infancy. Defining the relationship with COM advisors required time and frequent communication. Given that this podcast was a novel undertaking, there was little national precedent for empowering students’ voices while holding fast to expert review and professionalism standards. A method of graduated autonomy facilitated the team’s close contact with key podcast advisors at pivotal stages without squelching authentic student voices. Due to privacy considerations regarding academic performance, the recruitment of student guests mostly relied on preexisting social connections rather than the rigid, widespread screening of candidates. Although this may have weakened the overall credibility of the curricular success–related and USMLE-related episodes, this method ensured that there was good rapport between hosts and guests, which substantially contributed to the overall quality of conversations. Despite continually striving for excellent audio quality, finances and production naivety were frequent barriers to improvement. Funding provided by the Medical Student Association was appreciated, but social distancing guidelines generated additional costs during the transition to web-based hosting. The podcast team was able to overcome many technical barriers by consulting with seasoned podcasters in academic medicine and reading literature published on podcasting best practices [1,5]. Understanding listenership trends and preferences proved difficult, although frequent anecdotal feedback provided some limited insight.

To better understand podcast efficacy and listeners’ perceptions, we aim to collect data from the COM student body as part of an ongoing needs assessment. The literature regarding educational podcast creation is sparse, especially literature that pertains to the medical student population. Research is needed to understand the perceived benefits of medical education podcasts, their efficacy as teaching tools, and best practices for podcast production.

As medical education transitions from print formats to digital formats [17], medical student–run podcasts represent novel tools for supporting medical students, leading the student community into the future of medicine, and fostering professional identity formation. Podcasts are widely accessible and convenient for listeners, and podcast creators can publish content with lower barriers of entry compared to those of other forms of published content. Although school-specific podcasts may exclude a national audience, they enhance the depth of the sense of community among local students, clinicians, and educators. Medical schools should consider supporting student podcast initiatives to allow for near-peer mentoring, augment the community, facilitate professional identity formation, and prepare the rising physician workforce for the technological frontier of medical education and practice.

## Abbreviations

COM: College of Medicine
USMLE: US Medical Licensing Examination
